# Histogram analysis of DTI-derived indices reveals pontocerebellar degeneration and its progression in SCA2

**DOI:** 10.1371/journal.pone.0200258

**Published:** 2018-07-12

**Authors:** Mario Mascalchi, Chiara Marzi, Marco Giannelli, Stefano Ciulli, Andrea Bianchi, Andrea Ginestroni, Carlo Tessa, Emanuele Nicolai, Marco Aiello, Elena Salvatore, Andrea Soricelli, Stefano Diciotti

**Affiliations:** 1 “Mario Serio” Department of Experimental and Clinical Biomedical Sciences, University of Florence, Florence, Italy; 2 Department of Electrical, Electronic, and Information Engineering “Guglielmo Marconi”, University of Bologna, Cesena, Italy; 3 Unit of Medical Physics, Pisa University Hospital “Azienda Ospedaliero-Universitaria Pisana”, Pisa, Italy; 4 Neuroradiology Unit, Azienda Ospedaliero Universitaria Careggi, Florence, Italy; 5 Department of Radiology and Nuclear Medicine, Versilia Hospital, AUSL 12 Viareggio, Lido di Camaiore (Lu), Italy; 6 IRCSS Fondazione SDN, Naples, Italy; 7 Department of Neurological Sciences, University of Naples Federico II, Naples, Italy; 8 University of Naples Parthenope, Naples, Italy; University of Minnesota, UNITED STATES

## Abstract

**Purpose:**

To assess the potential of histogram metrics of diffusion-tensor imaging (DTI)-derived indices in revealing neurodegeneration and its progression in spinocerebellar ataxia type 2 (SCA2).

**Materials and methods:**

Nine SCA2 patients and 16 age-matched healthy controls, were examined twice (SCA2 patients 3.6±0.7 years and controls 3.3±1.0 years apart) on the same 1.5T scanner by acquiring T_1_-weighted and diffusion-weighted (b-value = 1000 s/mm^2^) images. Cerebrum and brainstem-cerebellum regions were segmented using FreeSurfer suite. Histogram analysis of DTI-derived indices, including mean diffusivity (MD), fractional anisotropy (FA), axial (AD) / radial (RD) diffusivity and mode of anisotropy (MO), was performed.

**Results:**

At baseline, significant differences between SCA2 patients and controls were confined to brainstem-cerebellum. Median values of MD/AD/RD and FA/MO were significantly (p<0.001) higher and lower, respectively, in SCA2 patients (1.11/1.30/1.03×10^−3^ mm^2^/s and 0.14/0.19) than in controls (0.80/1.00/0.70×10^−3^ mm^2^/s and 0.20/0.41). Also, peak location values of MD/AD/RD and FA were significantly (p<0.001) higher and lower, respectively, in SCA2 patients (0.91/1.11/0.81×10^−3^ mm^2^/s and 0.12) than in controls (0.71/0.91/0.63×10^−3^ mm^2^/s and 0.18). Peak height values of FA and MD/AD/RD/MO were significantly (p<0.001) higher and lower, respectively, in SCA2 patients (0.20 and 0.07/0.06/0.07×10^−3^ mm^2^/s/year /0.07) than in controls (0.15 and 0.14/0.11/0.12/×10^−3^ mm^2^/s/year /0.09). The rate of change of MD median values was significantly (p<0.001) higher (i.e., increased) in SCA2 patients (0.010×10^−3^ mm^2^/s/year) than in controls (-0.003×10^−3^ mm^2^/s/year) in the brainstem-cerebellum, whereas no significant difference was found for other indices and in the cerebrum.

**Conclusion:**

Histogram analysis of DTI-derived indices is a relatively straightforward approach which reveals microstructural changes associated with pontocerebellar degeneration in SCA2 and the median value of MD is capable to track its progression.

## Introduction

Spinocerebellar ataxia type 2 (SCA2) is the second more frequent SCA after SCA3, worldwide and entails a pattern of pontocerebellar degeneration at neuropathological examination [1]. MRI shows a variable combination of volume loss in T_1_-weighted images and symmetric areas of increased signal changes in T_2_-weighted images in the brainstem, cerebellar peduncles and cerebellum [2]. Moreover, quantitative diffusion-weighted imaging (DWI) and diffusion-tensor imaging (DTI) have proven to be useful in assessing microstructural changes of the brain tissue in SCA2 [2].

Brain DWI- or DTI-derived indices have proven to be correlated with severity of clinical deficit in several cross-sectional studies of patients with SCA2 [2–5], and may represent potential biomarkers of disease progression in longitudinal studies of neurodegenerative diseases including inherited or sporadic degenerative ataxias [6]. So far, this possibility has been explored through regions of interest (ROIs) [7,8] or voxel-wise tract based spatial statistics (TBSS) [9] analysis of DTI data [10,11]. While TBSS can provide whole brain and unbiased local information on the changes caused by disease, it is inherently restricted to white matter (WM) skeleton [9] assessment. Moreover, TBSS requires accurate normalization of maps of DTI-derived indices to a template [12] and is based on a number of assumptions which may not be satisfied, affecting the reliability of results [13]. In particular, small choices in the pre-processing pipeline may have a relevant effect on test-retest reliability, therefore influencing the power to detect change within a longitudinal study [14].

A histogram analysis of DWI- or DTI-derived indices of the whole or segmented brain structures to evaluate microstructural damage in degenerative ataxias (including SCA2) has been previously proposed [15]. This approach has a number of advantages as compared to ROI and TBSS methods, albeit it implies loss of spatial information on local changes. Notably, given that correction for multiple comparisons across several voxels or ROIs is not needed, a higher statistical power may be obtained. Also, histogram analysis can be extended to gray matter (GM) regions. Finally, in principle, analysis of normalized histograms can be performed without coregistration of maps of diffusion indices to a template. To date, there is only one longitudinal study that used histogram analysis of DTI-derived indices of the whole brain and whole WM in a neurodegenerative disorder, namely, Huntington disease [16].

In this longitudinal study, we carried out, in SCA2, histogram analysis of several DTI-derived indices of the segmented cerebrum and brainstem-cerebellum, including both WM and GM, in order to: 1) investigate whether such a relatively straightforward approach has the potential to reveal and track progression of microstructural damage; 2) preliminarily assess if the rate of change of histogram metrics of DTI-derived indices correlates with clinical deterioration.

## Materials and methods

### Subjects

We examined 9 patients (3 women and 6 men; age 48.7±12.9 years, mean ± standard deviation) with a genetically determined SCA2. They gave informed written consent to participate in this longitudinal study which was approved by the Local Ethics Committee of the Careggi University Hospital of Florence, Italy. Diagnosis was based on a number of triplet repeats expansions ≥ 34 CAG on one allele, and the mean number of abnormal triplets was 40.6±1.4 [17]. All patients underwent MRI twice, 3.6 ± 0.7 (mean ± standard deviation) years apart (range 2.2–4.0 years), on the same scanner by using the same acquisition protocol. The same clinician (A.G.) evaluated every patient by computing the duration of symptoms and signs at baseline MRI examination and by assessing the neurological deficit using the Inherited Ataxia Clinical Rating Scale (IACRS) [18] and the International Cooperative Ataxia Rating Scale (ICARS) [19] at both baseline and follow-up MRI examination. In the IACRS, signs and symptoms related to ataxia, but also pyramidal tract dysfunction and impaired vibration or position sense, which are frequently observed in SCA2, are semi-quantitatively assessed using a 0–38 score scale (with 38 corresponding to maximum deficit). In the ICARS, only cerebellar functions are semi-quantitatively assessed using a 0–100 score scale (with 100 corresponding to maximum deficit). At the time of baseline MRI, the disease duration since clinical onset in SCA2 patients was 12.8±7.3 (mean ± standard deviation) years (range 2–23 years), IACRS score was 17.2±4.3 (mean ± standard deviation) (range 9–25) and ICARS score was 39.7±14.3 (mean ± standard deviation) (range 15–54). Pyramidal signs were present in 3 of 9 patients, whereas no patient showed extra-pyramidal signs. At the time of follow up, the IACRS score was 21.3 ± 6.1 (mean ± standard deviation) (range 14–31) and ICARS score was 44.3 ± 14.5 (mean ± standard deviation) (range 19–62) and no additional patient showed pyramidal or extra-pyramidal signs.

We recruited as controls sixteen age- and gender-matched healthy subjects (7 women and 9 men; age 50.3±18.8 years, mean ± standard deviation) who had no familial or personal history of neurologic or psychiatric dysfunction and a normal neurological examination at baseline and follow-up. They provided an informed written consent to participate in the study. They underwent MRI twice, 3.3 ± 1.0 (mean ± standard deviation) years apart (range 1.9–4.7 years), by using the same scanner and acquisition protocol used for examination of SCA2 patients.

### MRI examination

A 1.5 T MRI scanner (Philips Intera, Best, The Netherlands) equipped with 33 mT/m maximum gradient strength and 6-channel phased-array head coil was utilized for baseline and follow-up MRI examinations in all patients and controls. After the scout, sagittal 3D T_1_-weighted turbo gradient echo [repetition time (TR) = 8.1 ms, echo time (TE) = 3.7 ms, flip angle = 8°, inversion time = 764 ms, field of view (FOV) = 256 mm × 256 mm, matrix size = 256×256, 160 contiguous slices, slice thickness = 1 mm] images were acquired. In addition, axial diffusion-weighted images were obtained with a single-shot echo-planar imaging sequence (TR = 9394 ms, TE = 89 ms, FOV = 256 mm × 256 mm, matrix size = 128×128, 50 slices, slice thickness = 3 mm, no gap, number of excitations = 3). Diffusion sensitizing gradients were applied along 15 non-collinear and non-coplanar directions with b-value of 0 (b_0_ image) and 1000 s/mm^2^.

T_1_-weighted and diffusion-weighted images were visually evaluated by a neuroradiologist (A.G.) for the identification of artifacts before entering further image processing. After this visual quality control, all images were retained for further processing.

### Gray and white matter segmentation

**Fig 1:**
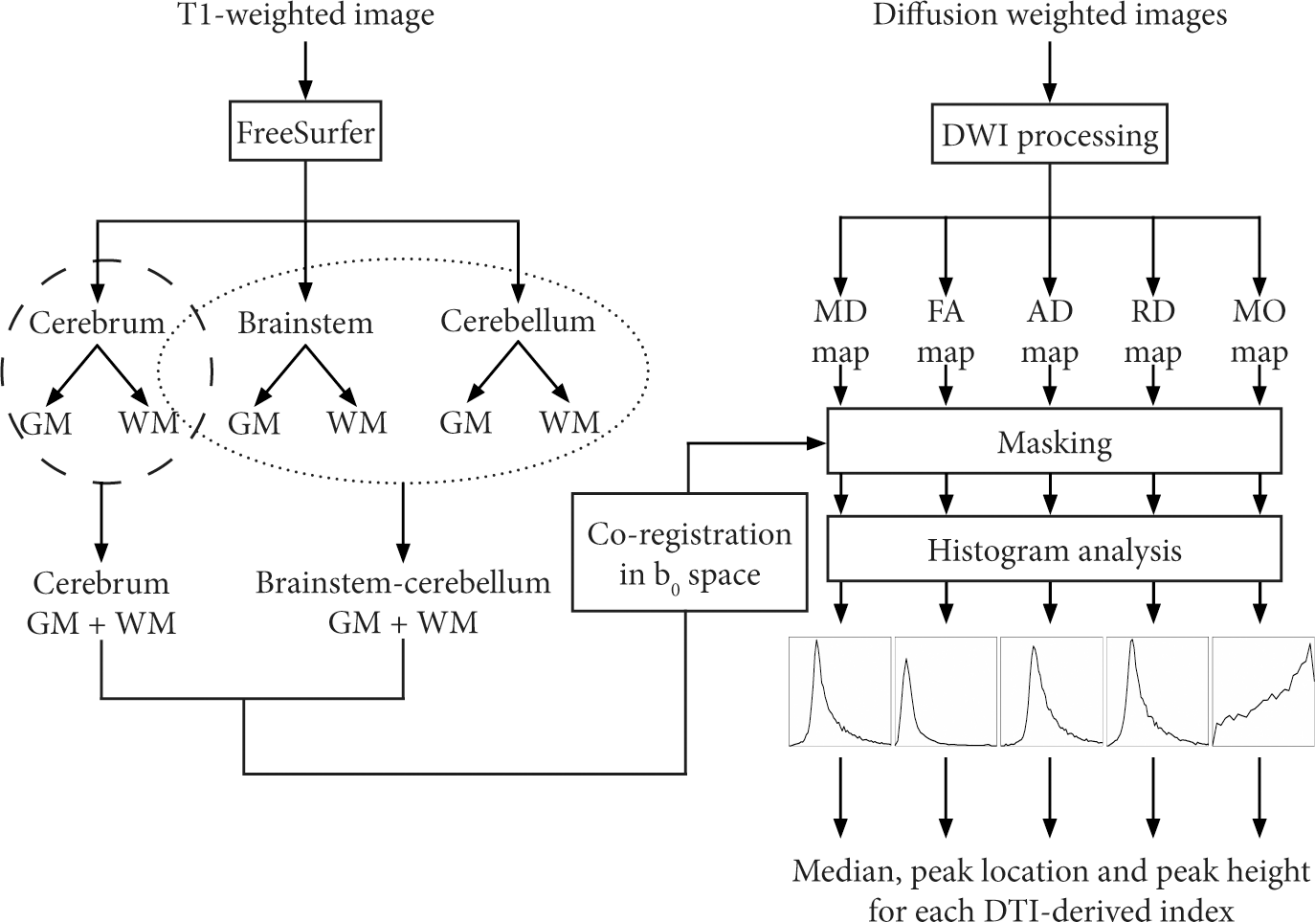
Processing pipeline for T_1_-weighted and diffusion-weighted images of a single subject.

Completely automated cortical reconstruction and segmentation of the subcortical WM of each subject were performed by means of T_1_-weighted images and *FreeSurfer* image analysis suite v. 5.3 (http://surfer.nmr.mgh.harvard.edu/) [20]. For each subject, the segmentation masks of GM/WM of cerebrum were merged in order to obtain a unique region mask for cerebrum, while the segmentation masks of GM/WM of brainstem and cerebellum were merged in order to obtain a unique region mask for brainstem-cerebellum (Fig 1). Cerebral segmentation and brainstem-cerebellum segmentation for a representative SCA2 patient are shown in S1 Fig. More details about the *FreeSurfer* procedures are described in the S1 Appendix.

### DTI processing

Diffusion-weighted images were corrected for head motion and eddy current distortions using FDT (FMRIB’s Diffusion Toolbox 2.0; FMRIB, Oxford Center for Functional MRI of the Brain), part of FSL (FMRIB Software Library) version 5.0.8 [21]. Skull was removed using the FSL brain extraction tool (BET) [22]. The b-matrix was reoriented by applying the rotational part of the affine transformation employed in the head motion and eddy current correction procedure [23]. Then, using the RESTORE diffusion tensor estimation [24] implemented in the CAMINO software package [25], a tensor model was fitted to processed DWI data. The diffusion tensor is described by three eigenvectors and relevant eigenvalues λ_1_, λ_2_ and λ_3_ [26]. In particular, the eigenvalues represent the diffusion coefficients of water molecules along the directions of the respective eigenvectors. Moreover, for gray matter and anisotropic white matter, the principal eigenvector individuates the direction along which water diffusivity is maximum and preferential direction of propagation of fiber bundles, respectively. Various rotationally invariant DTI indices can be derived from the eigenvalues of the diffusion tensor such as mean diffusivity (MD), fractional anisotropy (FA) and mode of anisotropy (MO). In particular, MD is proportional to the trace of the diffusion tensor and quantifies water molecules diffusivity independently of direction. FA (dimensionless) is the normalized standard deviation of the eigenvalues and measures the degree of water diffusion anisotropy, ranging from 0 (isotropic diffusion) to 1 (completely anisotropic diffusion). MO is a dimensionless measure of anisotropy type, ranging from -1 to +1: negative MO values describe planar anisotropy (i.e., two large and one small eigenvalue, as observed for instance in regions of crossing fiber bundles), whereas positive MO values indicate linear anisotropy (i.e., one large and two small eigenvalues, as observed for instance in major fiber bundles) [27,28]. Also, AD (i.e., the largest eigenvalue) quantifies the amount of water diffusivity along the direction of the principal eigenvector. RD is the average of the medium and smallest eigenvalues and quantifies water diffusivity in the plane perpendicular to the principal eigenvector. DTI-derived indices are proven to be sensitive to brain tissue microstructure, which is characterized by various factors including cell and axonal density/size, membrane permeability and integrity, fiber orientation dispersion and myelin sheath. Accordingly, the pattern of variation of DTI-derived indices can provide information about the microstructural changes underlying various brain diseases [29–31].

In this study, the DTI-derived indices of MD, FA, AD, RD and MO were estimated using DTI-TK version 2.3.1 [32], FSL tools and in-house Bash shell scripts.

### Histogram analysis

For each subject, the cerebrum and brainstem-cerebellum segmentations were converted from the *FreeSurfer* space back to the native anatomical space, and the T_1_-weighted image in the native space was co-registered to the b_0_ image using the 12 degrees of freedom affine transformation implemented in FSL FLIRT (FMRIB’s Linear Image Registration Tool) [33]. This affine transformation was then applied to cerebrum and brainstem-cerebellum segmentation (Fig 1).

**Fig 2:**
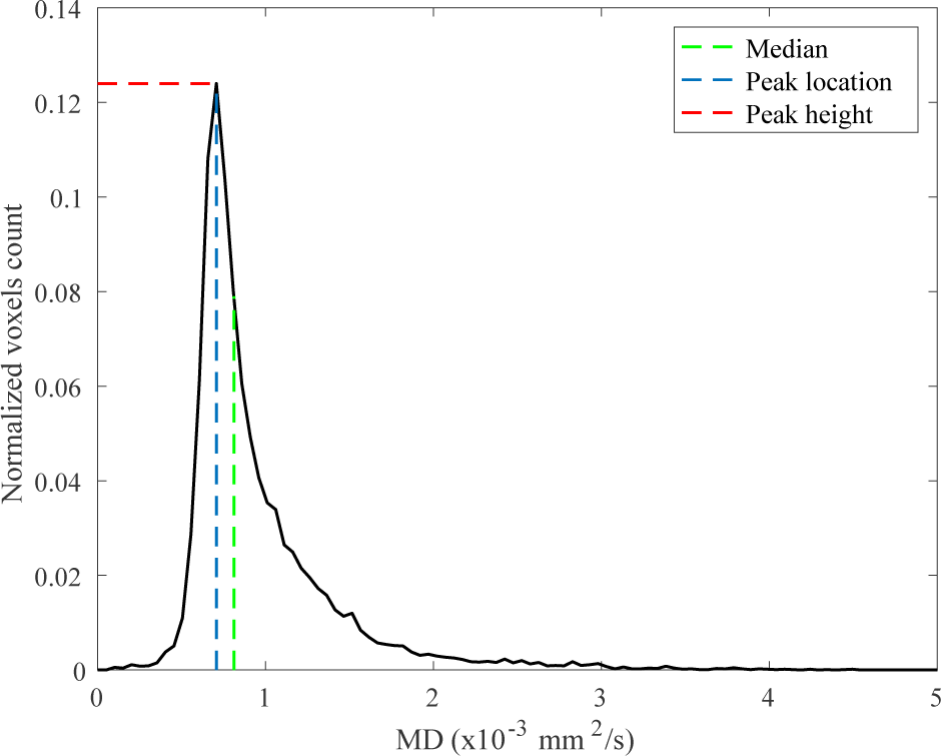
Example of histogram showing the 3 three histogram metrics we used: The median value, the peak location and peak height.

For each cerebrum and brainstem-cerebellum segmentation in the b_0_ space, the histogram (normalized over the total number of voxels) of MD/FA/AD/RD/MO was computed. The normalization allows to correct for individual differences in brain size. In this study, we used three histogram metrics of DTI-derived indices: the median value, the peak location and peak height (Fig 2). The median is the value of the DTI-derived index (i.e., MD, FA, AD, RD or MO) that divides the higher half of the data sample from the lower half. The peak location is the mode of the histogram, i.e. the most frequent value assumed by the DTI-derived index; the value of the histogram assumed at the peak location is the histogram peak height which is the maximum value of the histogram.

### Data analysis

At baseline, differences in histogram metrics of DTI-derived indices between SCA2 patients and control subjects were assessed through the non-parametric Mann Whitney test. For each histogram metric, the rate of change (i.e., the ratio between the change over time and the time-to-follow-up) was computed. Then, the non-parametric Mann Whitney test was employed in order to assess any difference in rate of change between SCA2 patients and control subjects. In SCA2 patients, the Pearson coefficient was employed in order to assess any linear correlation between the rate of change of histogram metrics of DTI-derived indices and clinical data (i.e., number of triplets in the longer allele, disease duration at baseline, clinical progression as assessed by the rate of change in IACRS and ICARS). For each test, the Holm-Bonferroni correction for multiple comparisons was applied (significance threshold of 0.05), in order to control the family wise error rate. Finally, in order to evaluate the sensitivity to change of ICARS, IACRS and histogram metrics of those DTI-derived indices showing rate of change significantly different between SCA2 patients and controls, we used the standardised response mean (SRM).

## Results

The descriptive statistics of histogram metrics of DTI-derived indices are reported in Table 1.

**Table 1:** Histogram metrics of DTI-derived indices in control subjects and SCA2 patients. Median (interquartile range) data are reported. MD/AD/RD median and peak location are expressed in ×10^−3^ mm^2^/s, while FA/MO median and peak location are unitless.

	Baseline		Rate of change	
	Controls	SCA2 patients	Controls	SCA2 patients
**Cerebrum**				
MD median	0.81 (0.02)	0.82 (0.03)	0.002 (0.005)	0.004 (0.007)
MD peak location	0.76 (0.00)	0.76 (0.05)	0.000 (0.000)	0.000 (0.000)
MD peak height	0.14 (0.02)	0.13 (0.03)	-0.001 (0.003)	-0.002 (0.003)
FA median	0.21 (0.02)	0.21 (0.01)	-0.001 (0.004)	-0.001 (0.003)
FA peak location	0.09 (0.00)	0.09 (0.00)	0.000 (0.000)	0.000 (0.004)
FA peak height	0.11 (0.01)	0.10 (0.01)	0.001 (0.002)	0.000 (0.001)
AD median	1.05 (0.05)	1.06 (0.03)	0.002 (0.007)	0.003 (0.005)
AD peak location	0.96 (0.05)	1.01 (0.01)	0.000 (0.013)	0.000 (0.000)
AD peak height	0.1 (0.02)	0.09 (0.01)	-0.001 (0.003)	-0.001 (0.003)
RD median	0.69 (0.02)	0.71 (0.02)	0.001 (0.005)	0.004 (0.008)
RD peak location	0.63 (0.05)	0.66 (0.01)	0.000 (0.000)	0.000 (0.000)
RD peak height	0.11 (0.01)	0.11 (0.02)	0.000 (0.002)	-0.001 (0.002)
MO median	0.37 (0.03)	0.35 (0.03)	0.001 (0.004)	-0.003 (0.005)
MO peak location	0.92 (0.00)	0.92 (0.00)	0.000 (0.000)	0.000 (0.000)
MO peak height	0.09 (0.00)	0.09 (0.00)	0.000 (0.001)	0.000 (0.001)
**Brainstem-cerebellum**				
MD median	0.8 (0.03)*	1.11 (0.12)*	-0.003 (0.006)*	0.010 (0.014)*
MD peak location	0.71 (0.00)*	0.91 (0.1)*	0.000 (0.000)	0.000 (0.026)
MD peak height	0.14 (0.02)*	0.07 (0.02)*	0.001 (0.006)	-0.001 (0.002)
FA median	0.20 (0.02)*	0.14 (0.02)*	0.001 (0.006)	-0.001 (0.006)
FA peak location	0.18 (0.03)*	0.12 (0.00)*	0.000 (0.000)	0.000 (0.010)
FA peak height	0.15 (0.02)*	0.2 (0.03)*	-0.001 (0.005)	-0.002 (0.007)
AD median	1.00 (0.05)*	1.3 (0.12)*	-0.002 (0.011)	0.010 (0.018)
AD peak location	0.91 (0.05)*	1.11 (0.18)*	0.000 (0.006)	0.000 (0.051)
AD peak height	0.11 (0.02)*	0.06 (0.01)*	0.001 (0.004)	-0.001 (0.002)
RD median	0.7 (0.03)*	1.03 (0.11)*	-0.004 (0.008)	0.011 (0.018)
RD peak location	0.63 (0.05)*	0.81 (0.13)*	0.000 (0.006)	0.000 (0.016)
RD peak height	0.12 (0.02)*	0.07 (0.01)*	0.000 (0.005)	-0.001 (0.002)
MO median	0.41 (0.11)*	0.19 (0.13)*	-0.003 (0.029)	-0.006 (0.023)
MO peak location	0.92 (0.00)	0.92 (0.00)	0.000 (0.000)	0.000 (0.025)
MO peak height	0.09 (0.02)*	0.07 (0.01)*	-0.001 (0.006)	-0.002 (0.004)

*significant differences (p<0.001) between controls and SCA2 patients after Holm-Bonferroni correction for multiple comparisons.

**Fig 3:**
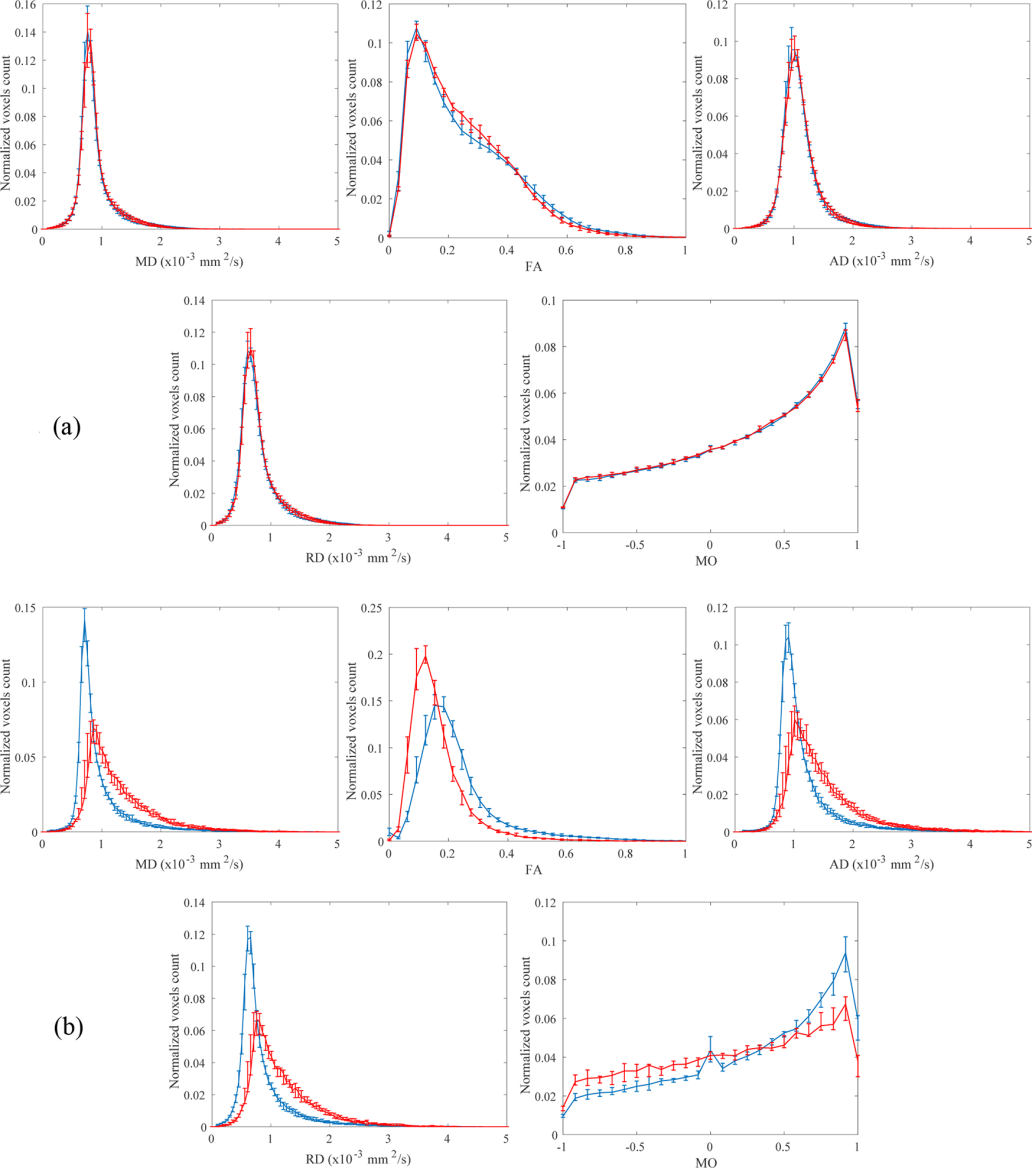
The histograms (median with the interquartile range) of control subjects (blue line) and SCA2 patients (red line) groups of DTI-derived indices of cerebrum (a) and cerebellum-brainstem (b) are shown. The bin width is 0.05×10^−3^ mm^2^/s for MD/AD/RD, 0.03 for FA and 0.08 for MO. MD/AD/RD (median, peak location) and FA/MO (median, peak location) are reported as 10^−3^ mm^2^/s and unitless, respectively.

Histograms of DTI-derived indices of control subjects and SCA2 patients groups are shown in Fig 3.

At baseline, significant differences between SCA2 patients and controls were confined to brainstem-cerebellum. In particular, median values of MD/AD/RD and FA/MO were significantly (p<0.001) higher and lower, respectively, in SCA2 patients than controls (Table 1). Also, peak location values of MD/AD/RD and FA were significantly (p<0.001) higher and lower, respectively, in SCA2 patients than controls (Table 1). Peak height values of FA and MD/AD/RD/MO were significantly (p<0.001) higher and lower, respectively, in SCA2 patients than controls (Table 1).

The rate of change of MD median values was significantly (p<0.001) higher (i.e., increased) in SCA2 patients than controls in the brainstem-cerebellum (Table 1). No other significant difference in rates of change of histogram metrics of MD and other DTI-derived indices between SCA2 patients and controls was found.

No significant correlation between the rate of change of histogram metrics of DTI-derived indices and disease duration, number of triplets or rate of change of the IACRS or ICARS scores was observed.

The SRM of the median MD in cerebellum-brainstem was intermediate (SRM = 1.2) between that of ICARS (SRM = 1.0) and IACRS (SRM = 1.3).

## Discussion

SCA2 belongs to the polyglutamine diseases group that comprises nine neurodegenerative conditions which share abnormal expansion of a CAG triplet in the coding region of the mutated gene as fundamental pathogenetic mechanism [1]. In particular, SCA2 is inherited as an autosomal dominant tract and involves expansion in excess of 32 CAG repeats in the disease gene Ataxin-2. The expanded Ataxin-2 mainly targets Purkinje cell in the cerebellum and several pontine neurons, localizes to RNA containing stress granules, is associated with the endoplasmic reticulum/Golgi fraction, and has a presumable role in cytoplasmic RNA-related functions [1].

SCA2 is a neurodegenerative disease that is progressive and ultimately fatal. So far, the therapeutic window to slow, halt and hopefully reverse the degenerative process in SCA2 has not been established yet. Identification and comparison of reliable and sensitive markers of disease progression potentially serving as primary or surrogate markers in future trials, including quantitative MRI [6], represent hence active areas of research. Several clinical measurements of disease progression in SCA2 and in ataxias in general have been proposed [18,19]. However, all clinical scales are subjective and are deemed not to be fairly sensitive to disease progression [34].

SCA2 shares with other SCAs and multi system atrophy (MSA) a pattern of pontocerebellar atrophy at neuropathology [1] and MRI examination [2]. In particular, the microscopic brain examination shows widespread neuronal loss in the GM which is earlier and more prominent in the cerebellar cortex and pontine nuclei, the midbrain and medulla [1]. WM damage in SCA2 is remarkable and is characterized by loss of myelinated fibers and gliosis that involve the transverse fibers of the pons, cerebellum, the middle and inferior cerebellar peduncles, the medial lemnisci and trigeminal tracts, the fascicule gracilis and cuneatus and the spinocerebellar tracts [1]. SCA2 can actually represent a prototype of both polyglutamine disease and of pontocerebellar degeneration.

The results of this study suggest that histogram metrics of five DTI-derived indices may be a useful tool to reveal microstructural changes associated with the brainstem-cerebellum degeneration in SCA2 patients and that one of these indices, namely MD, is able to track modification over time of the microstructural changes candidating this index as a potential biomarker of disease progression.

The modifications of the histogram metrics and distribution of DTI-derived indexes in SCA2 patients, as compared to control subjects, at baseline (and follow-up) are in line with the general features of DTI changes in neurodegenerative diseases [30]. In fact, both the increase (implying a more pronounced peak) of voxels exhibiting lower values (implying a shift to the left), in case of FA, and the decrease (implying a less pronounced peak) of voxels exhibiting variably higher values (implying a shift to the right), in case of MD/AD/RD, reflect tissue loosening. In case of MO, the peak height is reduced, and the histogram values are higher for negative values of MO, meaning that the mode of anisotropy tends to change from linear anisotropy (MO = 1) to planar anisotropy (MO = -1).

Notably, while increased MD and RD and decreased FA of the affected nervous tissue are generally observed in patients with various neurodegenerative disorders as compared to healthy controls [30], AD and MO can have a dual (increase/decrease) pattern of change in the same patient group [10,15]. In the present study, at baseline we observed higher median and peak location values of AD in SCA2 patients as compared to control subjects. This is consistent with previous data in Friedreich’s ataxia and Huntington disease [16,35,36], suggesting that neurodegeneration may be associated with increased AD. The pathophysiological interpretation of this phenomenon is not established, but some studies have hypothesized an increased extracellular water content secondary to atrophy of the WM fibers, which would allow faster water molecule movement parallel to axons, alterations of axonal water content and flux secondary to breakdown or accumulation of certain constituents of the cytoskeleton and a possible contribution of glial alterations [36].

The capability of histogram analysis of one of the five DTI-derived indexes, namely MD, to track progression of pontocerebellar degeneration integrates previous findings [10] and further supports the potential of DTI in assessing longitudinal changes in SCA2. In a previous TBSS study [10], only AD and MO indices showed longitudinal changes in SCA2 patients as compared to control subjects. In particular, AD changes were significantly greater (i.e., increased) in patients with SCA2 than in controls in WM tracts of the right cerebral hemisphere and corpus callosum, but not in the brainstem or cerebellum, whereas longitudinal MO changes were significantly lower (i.e., decreased) in patients with SCA2 than controls in hemispheric cerebral WM, corpus callosum, internal capsules, pons and left cerebellar peduncles, cerebral peduncles and WM of the left paramedian vermis. In this study, longitudinal changes of the median values of MD were significantly greater (i.e., increased) in patients with SCA2 than controls in the whole brainstem-cerebellum. Importantly, this capability of MD to reveal progression of neurodegeneration in SCA2, on the one hand, is consistent with an increase of tissue loosening and, on the other hand, replicates findings obtained with ROIs or TBSS analyses in other neurodegenerative disorders, including MSA, Huntington disease and Alzheimer disease [8,30].

In this study, we preliminary explored whether the rate of changes of histogram metrics of DTI-derived indices correlated with disease duration and the rate of change of the clinical deficit. In agreement with a previous TBSS study [10], no significant correlation was observed. However, this lack of significant correlation could indicate that DTI-derived indices, which strictly depend on tissue microstructure, are more sensitive to disease progression than clinical measurements, albeit this deserves further investigations in greater sample sizes. Nonetheless, our results support the hypothesis that DTI-derived indices, in particular MD, may constitute potential non-invasive and sensitive biomarkers of disease progression in degenerative ataxias [6].

The SRM of the two clinical scales in our SCA2 patients were comparable to those of other clinical scales in a morphometric study of SCA1, SCA3 and SCA6 [37]. The slightly higher value of IACRS presumably reflect inclusion of additional non-cerebellar deficits in this scale and the degeneration of additional neural structures beside the cerebellum in SCA2 [1]. In our study, the SRM of the median MD in cerebellum-brainstem was intermediate between the two clinical scales, but lower than those reported for several morphometric features in the above study of SCA1, SCA3 and SCA6 [37]. This might imply a relatively lower sensitivity of the microstructure changes revealed by DTI as compared to those of morphometry or reflect disease specific differences. However, this issue deserves to be addressed in future studies.

We recognize some limitations of this study. First, due to hardware and software constraints, we used a DTI acquisition protocol with only 15 diffusion weighting directions, resulting in a potential reduction of statistical power of the study. However, given that our histogram analysis includes mostly gray matter with isotropic diffusion and white matter regions with low/moderate diffusion anisotropy (i.e., FA < 0.6) (see Fig 3), the use of 15 diffusion weighting directions, albeit not optimal, can be assumed to be sufficient to minimize the rotational variance due to noise in the estimation of DTI-derived indices of MD and FA [38]. Second, admittedly, we arbitrarily decided to re-scan the patients (and controls) only once after a relatively long period of time since basal MRI. However, this interval is clinically reasonable (and tentatively adequate for therapeutic trials) and was justified, on the one hand, by the lack of any clue about the minimum time required to observe changes of DTI-derived indices in SCA2 and inherited pontocerebellar degenerations and, on the other hand, by the small number of patients in our cohort that might have entailed a possible beta error if they were re-scanned in a shorter period. Additional time points and greater sample sizes might enable a more accurate assessment of the dynamic of the neurodegenerative process. Finally, we performed a single center study, while using of DTI-derived indices as biomarker in rare diseases such as SCA2 would greatly benefit of multi-center studies. In this regard, histogram analysis of DTI-derived indices of segmented brain, given its simplicity and high reproducibility [39], might be adopted in future multi-centric studies such as the ENIGMA-Ataxia project (enigma@ini.usc.edu).

In conclusion, histogram analysis of five DTI-derived indices including MD, FA, AD, RD and MO is a relatively straightforward approach that is capable to reveal WM and GM microstructural changes associated with pontocerebellar degeneration in SCA2. Moreover, the median value of MD in the brainstem-cerebellum is capable to track progression of pontocerebellar degeneration. Histogram metrics of DTI-derived indices could hence serve as biomarkers of disease status and progression in SCA2.

## Supporting information

S1 Fig Example of gray/white matter segmentation of cerebrum (green) and brainstem-cerebellum (red) in one representative SCA2 patient. (TIF)

S1 Appendix Gray and white matter segmentation. (DOC)
