# Supplementary figures and images for: Histogram analysis of DTI-derived indices reveals pontocerebellar degeneration and its progression in SCA2
Source: PLoS One. 2018 Jul 12;13(7):e0200258. doi: 10.1371/journal.pone.0200258 (PMC6042729; doi:10.1371/journal.pone.0200258)

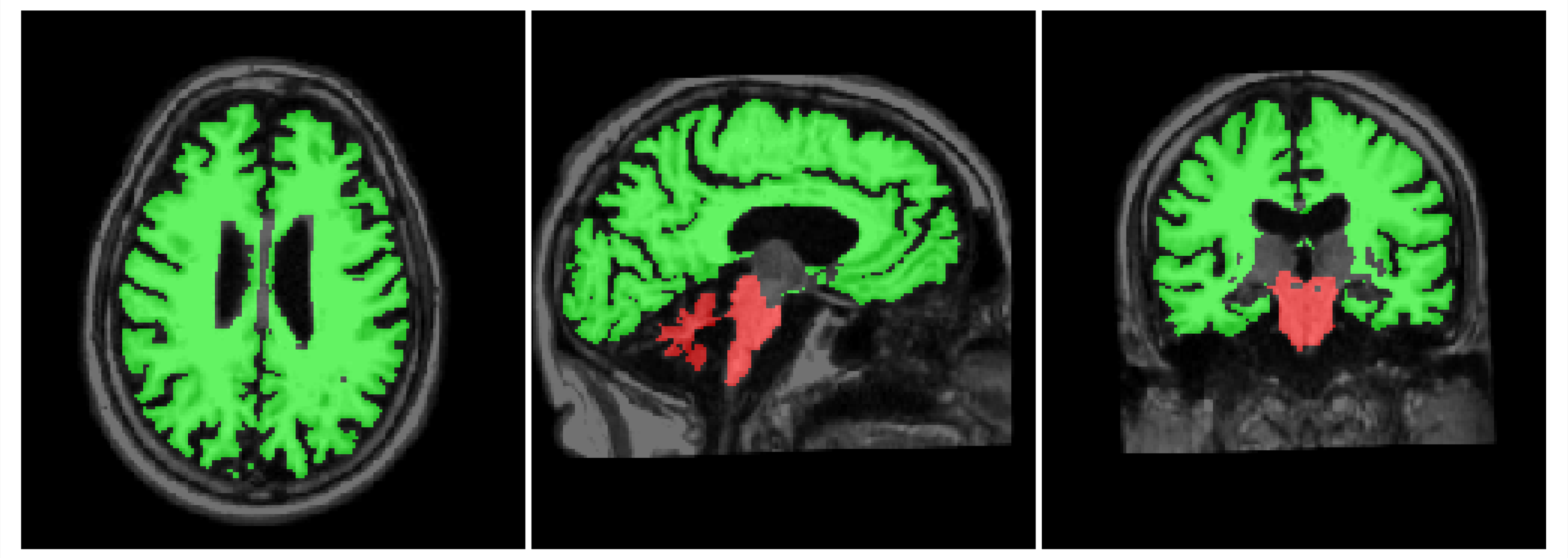

Supplement: S1 Fig — (TIF) [file pone.0200258.s001.tif]
