# Supplementary material for: Histogram analysis of DTI-derived indices reveals pontocerebellar degeneration and its progression in SCA2
Source: PLoS One. 2018 Jul 12;13(7):e0200258. doi: 10.1371/journal.pone.0200258 (PMC6042729; doi:10.1371/journal.pone.0200258)
Supplement: S1 Appendix — (DOC) [file pone.0200258.s002.doc]

**S1 Appendix**

**Gray and white matter segmentation**

Completely automated cortical reconstruction and segmentation of the subcortical WM of each subject by means of *FreeSurfer* image analysis includes removal of non-brain tissue using a hybrid watershed/surface deformation procedure, automated Talairach transformation, segmentation of the subcortical WM and deep GM volumetric structures, intensity normalization, tessellation of the GM/WM boundary, automated topology correction [1] and surface deformation, following intensity gradients to optimally place the GM/WM and GM/cerebrospinal fluid borders at the location where the greatest shift in intensity defines the transition to the other tissue class. Once the cortical models are complete, a number of deformable procedures can be performed for further data processing and analysis, including surface inflation, registration to a spherical atlas which is based on individual cortical folding patterns to match cortical geometry across subjects and creation of a variety of surface based data including maps of curvature and sulcal depth.

Given that the gross neuropathological appearance of SCA2 is a pontocerebellar atrophy with loss of bulk and predominant involvement of brainstem and cerebellum [2, 3] the *FreeSurfer* suite was employed to carry out also automated segmentation of GM/WM of each subject’s brainstem and cerebellum.

Cortical reconstruction and GM/WM surfaces were manually inspected for defects. The correction techniques suggested by *FreeSurfer* developers, including editing of brain/WM masks as well as adding control points and re-running of the *FreeSurfer* pipeline (<https://surfer.nmr.mgh.harvard.edu/fswiki/FsTutorial/TroubleshootingData>), were applied. All views (coronal, sagittal and axial) were used to confirm segmentation errors. The manual editing and re-running was carried out up to 2 times to assure that all defects were corrected [4]. Brainstem and cerebellum were manually edited using a free-hand pencil using the *Freeview* utility, part of *FreeSurfer* suite.

**References**

1. Fischl B, Liu A, Dale AM (2001) Automated manifold surgery: constructing geometrically accurate and topologically correct models of the human cerebral cortex. IEEE Trans Med Imaging 20: 70-80.

2. Mascalchi M, Vella A (2012) Magnetic resonance and nuclear medicine imaging in ataxias. Handb Clin Neurol 103: 85-110.

3. Della Nave R, Ginestroni A, Tessa C, Salvatore E, De Grandis D, et al. (2008) Brain white matter damage in SCA1 and SCA2. An in vivo study using voxel-based morphometry, histogram analysis of mean diffusivity and tract-based spatial statistics. Neuroimage 43: 10-19.

4. McCarthy CS, Ramprashad A, Thompson C, Botti JA, Coman IL, et al. (2015) A comparison of FreeSurfer-generated data with and without manual intervention. Front Neurosci 9: 379.
